# RNA induces unique tau strains and stabilizes Alzheimer’s disease seeds

**DOI:** 10.1016/j.jbc.2022.102132

**Published:** 2022-06-11

**Authors:** Amy N. Zwierzchowski-Zarate, Aydé Mendoza-Oliva, Omar M. Kashmer, Josue E. Collazo-Lopez, Charles L. White, Marc I. Diamond

**Affiliations:** Center for Alzheimer’s and Neurodegenerative Diseases, Peter O’Donnell Jr. Brain Institute, University of Texas Southwestern Medical Center, Dallas, Texas, USA

**Keywords:** tau, tauopathy, Alzheimer disease, neurodegenerative disease, RNA, fibril, oligomer, protein self-assembly, AD, Alzheimer’s disease, FL, full length, FRET, fluorescence resonance energy transfer, RBP, RNA-binding protein

## Abstract

Tau aggregation underlies neurodegenerative tauopathies, and transcellular propagation of tau assemblies of unique structure, *i.e.,* strains, may underlie the diversity of these disorders. Polyanions have been reported to induce tau aggregation *in vitro*, but the precise trigger to convert tau from an inert to a seed-competent form in disease states is unknown. RNA triggers tau fibril formation *in vitro* and has been observed to associate with neurofibrillary tangles in human brain. Here, we have tested whether RNA exerts sequence-specific effects on tau assembly and strain formation. We found that three RNA homopolymers, polyA, polyU, and polyC, all bound tau, but only polyA RNA triggered seed and fibril formation. In addition, polyA:tau seeds and fibrils were sensitive to RNase. We also observed that the origin of the RNA influenced the ability of tau to adopt a structure that would form stable strains. Human RNA potently induced tau seed formation and created tau conformations that preferentially formed stable strains in a HEK293T cell model, whereas RNA from other sources, or heparin, produced strains that were not stably maintained in cultured cells. Finally, we found that soluble, but not insoluble seeds from Alzheimer’s disease brain were also sensitive to RNase. We conclude that human RNA specifically induces formation of stable tau strains and may trigger the formation of dominant pathological assemblies that propagate in Alzheimer’s disease and possibly other tauopathies.

Tau forms highly ordered assemblies, termed amyloids, that underlie Alzheimer’s disease (AD) and related tauopathies (1), which may progress based on trans-cellular propagation (2, 3). The fundamental origin of tauopathy is unknown, but systemic triggers include amyloid beta, trauma, and inflammation. Tau assemblies adopt faithfully self-replicating conformations in cells, termed strains. Strains may be propagated indefinitely in cell culture (4, 5) and induce specific patterns of neuropathology when inoculated into mouse models (6, 7). We have observed that certain strains faithfully transmit neuropathology as prions (4). However, so far, stable amplification and propagation of defined strains *in vitro* has proven difficult. It is unknown why tau forms unique strains, but the observation that a seed-competent tau monomer encodes limited strain ensembles (8) suggests that there may be very specific molecular inducers of different strains. Specific posttranslational modifications of insoluble tau are reported to correlate with neuropathological diagnosis (9), but it is unknown whether phosphorylation triggers the conversion of inert to seed-competent tau monomer in tauopathy. Recent work from our lab has failed to find any detected posttranslational modification of tau that correlates with this event (10).

Recombinant tau is thermostable and remarkably inert in solution. In the last ∼30 years, multiple inducers of tau fibril formation *in vitro* have been described (11), including polyanions such as heparin (12, 13), fatty acids (14), octadecyl sulfate (15), and RNA (16). Tau was initially described to bind RNA, which sequestered it and prevented spontaneous tubulin assembly (17). RNA has also been observed in association with tau tangles in brain samples (18, 19) and in association with induced tau aggregates in HEK293 cells (20). Because tau has multiple positively charged residues, especially lysines, it was logical to assume that polyanions trigger tau assembly formation *in vitro* by neutralizing charge interactions and somehow unfolding the protein (21, 22) to facilitate its self-assembly.

The development of cell lines that amplify tau seeds, termed biosensors, has transformed our ability to characterize prion-like activity of relatively small amounts of soluble tau species (23, 24). Biosensors are based on expression of full-length (FL) or repeat domain (RD)-segments of tau (with or without disease-associated mutations) fused to suitable fluorescent protein tags. They respond to exogenous tau seeds by forming thioflavin positive inclusions (4). Biosensor cell lines can indefinitely and faithfully propagate myriad tau strains (4, 5), suggesting innate mechanisms of specific replication. Strains derived from cells create specific, transmissible forms of neuropathology after inoculation in a transgenic mouse model (PS19) that expresses tau (1N4R) containing a disease-associated mutation (P301S) (4, 6). It is thus feasible to create and study tau strains induced *in vitro* and propagated in cultured cells. Given our interest in physiologic inducers of seed and strain formation, for which RNA seemed a plausible candidate, we investigated its role in this process.

## Results

### Tau binds polyA, polyC, and polyU RNA homopolymers

**Figure 1:**
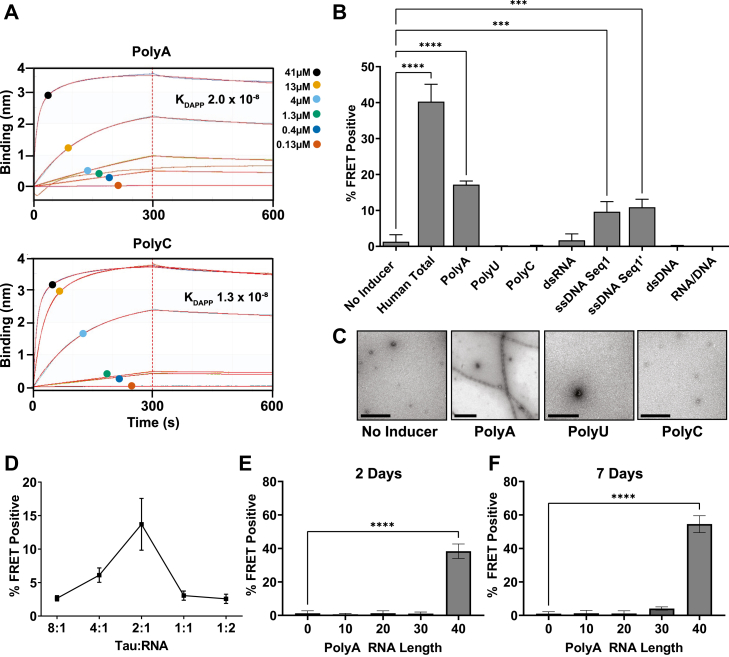
**Tau nonselectively binds RNA yet seeding and fibrilization are specific.***A*, BLI with tau immobilized onto AR2G biosensors and exposed to increasing concentrations of PolyA, or C RNA (half-log dilutions: 0.13 μM (*red*), 0.4 μM (*dark blue*), 1.3 μM (*green*), 4 μM (*light blue*), 13 μM (*orange*), and 41 μM (*black*)). Tau bound all RNA with similar avidity, association, and dissociation rates. Figure shows representative data of polyA or polyC RNA binding to BLI AR2G sensors loaded with tau. K_DAPP_ calculated as mean of three independent experiments. Binding (nm) refers to the wavelength perturbation in reflected light from the biosensor. *Red lines* represent curves fit to primary data. See Table 1 and Fig. S1 for more data on these experiments. *B*, tau monomer incubated 24 h with nucleic acid before transduction of v2L biosensors. Intracellular aggregation was quantified as % FRET positive *via* flow cytometry. Specific nucleic acids induced seed-competency in tau (human total RNA *p* < 0.0001, polyA RNA *p* < 0.0001, ssDNA Seq1 *p* = 0.0007, ssDNA Seq1′ *p* = 0.0002) while others did not (polyU RNA *p* = 0.98, polyC RNA *p* = 0.99, dsRNA *p* = 0.99, dsDNA *p* = 0.99, RNA/DNA *p* = 0.98). *p* values calculated with one-way ANOVA, post-hoc Dunnett’s multiple comparisons test. *C*, after 1 week with indicated inducer, tau was deposited on 400CF grids (4 μM) and stained with 2% uranyl acetate. Grids were scanned with TEM for fibrils, and representative images are shown. No fibrils were seen for no inducer, polyU, or polyC. Fibrils were observed only in conditions that induced seeding (polyA). Scale bar = 500 nm. *D*, tau monomer incubated with various molar ratios of polyA for 24 h before induction of biosensors. 2:1 Tau to RNA (8 μM:4 μM) ratio produced optimal seeding. *E* and *F*, seeding of tau following incubation with various lengths (nt) of polyA RNA for 2 (*E*) or 7 (*F*) days before transducing biosensors. >40 nt was required for tau seeding. (2 days *versus* 0, *p* = 0.97 10mer, *p* > 0.99 20mer, *p* = 0.99 30mer, *p* < 0.0001 40mer; 7 days *versus* 0, *p* = 0.99 10mer, *p* > 0.99 20mer, *p* = 0.15 30mer, *p* < 0.0001 40mer) *p* values calculated with one-way ANOVA, post-hoc Dunnett’s multiple comparisons test. All seeding data represents two experimental replicates, each performed in technical triplicate. These experiments are representative of similar studies performed 19 times overall for (B), and five times overall for (*D-F*). Error bars = S.D. ∗∗∗∗*p* < 0.0001, ∗∗∗*p* < 0.001.

We first tested whether homopolymers of RNA would differentially bind recombinant tau. We used 40-mers of adenine (A), cytidine (C), and uracil (U), omitting guanidine because of its difficulty to synthesize. We purified recombinant, FL tau monomer (2N4R) according to our prior methods (25), termed “tau” henceforth. We immobilized tau on amine reactive second-generation biolayer interferometry biosensors (ForteBio) and with exposure to increasing half-log concentrations of single-stranded RNA (0.13 μM, 0.4 μM, 1.3 μM, 4 μM, 13 μM, and 41 μM). We measured avidity based on changes in surface interferometry (Octet, ForteBio). Binding data were interpolated according to a 2:1 interaction model. PolyA, polyU, and polyC strands each bound tau with similar avidities in the nanomolar range, as did a randomly generated single strand of DNA (Figs. 1*A* and S1, Table 1).

**Table 1:** Affinity of tau for nucleic acid sequences

Inducer	K_DAPP_	k_on_ (M^−1^ s^−1^)	k_off_ (s^−1^)
polyA	20 nM	1.0 X 10^4^	1.4 X 10^−4^
polyU	254 nM	1.7 X 10^3^	4.2 X 10^−4^
polyC	13 nM	3.7 X 10^4^	4.5 X 10^−4^
ssDNA seq1	12 nM	2.8 X 10^4^	3.4 X 10^−4^

Abbreviations: K_DAPP_, apparent binding affinity; k_on_, association rate; k_off_, dissociation rate.

Results were calculated as the mean of three independent experiments.

### polyA induces tau seed formation *in vitro*

We next tested the ability of RNA homopolymers to induce tau fibril formation *in vitro*. We incubated tau (8 μM) in the presence of polyA, polyU, and polyC RNA (50 μg/ml, or 4 μM) for 24 h before measuring seeding with v2L biosensor cells that stably expressed the tau repeat domain with the P301S mutation fused to cerulean and mClover fluorescent proteins. After 48 h, we quantified intracellular aggregation by fluorescence resonance energy transfer (FRET) as per prior studies (23). PolyA generated seeding activity from tau monomer, while polyU and polyC did not (Fig. 1*B*). A polyU/polyA hybrid also failed to elicit seeding. Two single-stranded DNA homopolymers (polydA, polydT) did not induce seeding (Fig. S2*A*). Two randomly generated single-stranded, complementary DNA sequences of the same length and similar molecular weight as the RNA both effectively seeded, while the annealed double stranded complex did not, nor did an RNA/DNA complex of polyA hybridized with polydT (Fig. 1*B*).

To rule out kinetic differences in seed induction, we incubated tau and RNA for up to 2 weeks. While polyA induced seed-competent forms, polyC and polyU never did (Fig. S2, *A*–*C*). We observed fibril formation *via* transmission electron microscopy (TEM) that correlated with seeding activity after 48 h incubation (Fig. 1*C*). Thus, despite universal binding of RNA to tau, only certain single-stranded nucleotide sequences induced seed-competent conformations.

The optimal ratio of RNA:tau to induce seeding was 1:2 (Fig. 1*D*). We defined the minimal length of RNA necessary to induce seeding by incubating tau with increasing sizes of polyA ranging from 10 to 40 nucleotides (nt) for various time periods. At 48 h, the smallest length of RNA capable of inducing seed-competent tau to a significant degree was 40 nt (*p* < 0.0001, one-way ANOVA, post-hoc Dunnett’s multiple comparisons test) (Fig. 1*E*). After 7 days, we observed minimal increases in seed formation with 30 nt (4.1% FRET positive), which were still not significant compared to buffer control (*p* = 0.1451, one-way ANOVA, post-hoc Dunnett’s multiple comparisons test), while 40 nt seeding increased to 55% FRET positive.

### RNA stabilizes seeds and fibrils

**Figure 2:**
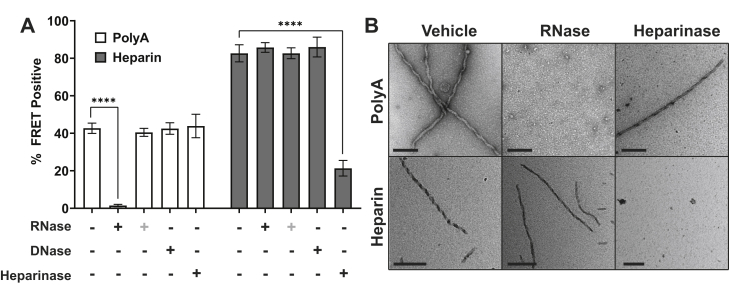
**Stabilization of tau fibrils and seeds *in vitro*.***A*, tau fibrils preformed with polyA RNA or heparin were treated with RNase (T1 and A, 316 U/μl and 3.2 mg/ml), DNase (I, 633 U/μl), or heparinase I (3797 U/ml) for 24 h before transduction of biosensors. (+) signifies RNase treatment added to fibrils at same time as seeding, with no pre-incubation. Seeds formed with polyA were sensitive to RNase treatment (*p* < 0.0001). RNase added directly to seeds at the time of seeding had no effect (*p* = 0.49). DNase and heparinase treatment did not affect seeds formed by polyA (*p* = 0.99 and 0.97). Seeds formed with heparin were sensitive to heparinase treatment (*p* < 0.0001), but not RNase or DNase (*p* = 0.09 RNase, *p* > 0.99 immediate RNase, *p* = 0.24 DNase). *p* values were calculated with one-way repeated measures ANOVA, post-hoc Dunnett’s multiple comparisons test, comparing control to enzyme treated conditions for both polyA and heparin fibrils. Seeding data represents two experimental replicates, each performed in technical triplicate. These experiments are representative of similar studies performed 15 times overall. Error bars = S.D. *B*, TEM 400CF grids containing tau (4 μM) stained with 2% UA. All grids were scanned; representative images are shown. Scale bar = 200 nm. Fibrils formed by polyA lose integrity after exposure to RNase, but not heparinase. Heparin fibrils were undetectable after heparinase treatment, but not RNase. FRET, fluorescence resonance energy transfer. ∗∗∗∗*p* < 0.0001.

Amyloid fibrils are thought to occupy a particularly stable, low-energy state (26), which might predict their persistence after inducers are removed. We tested this idea for RNA induction. We first formed tau fibrils by incubation with polyA RNA. We then exposed fibrils to a mixture of RNase A and RNase T1 for 24 h to degrade the RNA and tested the effect on seeding and fibril integrity. Tau seeds induced by RNA lost all seeding activity after RNase exposure, while incubation with DNase or heparinase had no effect (Fig. 2*A*). To exclude a direct effect of RNase on biosensor activity, we added RNase directly to the seeds without preincubation and detected no loss in seeding activity (Fig. 2*A*). We next used TEM to test fibril stability in the presence of nucleases. RNase eliminated all detectable fibrils, but not DNase or heparinase (Fig. 2*B*). Conversely, fibrils induced by heparin remained unchanged after DNase and RNase treatment but disassembled and lost 74% of seeding after incubation with heparinase (Fig. 2, *A* and *B*). Incomplete loss of seeding after removal of heparin is consistent with our prior observations that it converts tau monomer to a highly stable, seed-competent conformation (27, 28).

### RNA influences strain composition

Tau adopts multiple, faithfully propagated assembly structures that produce unique, transmissible patterns of neuropathology *in vivo*, termed strains (4, 6). Given that RNA sequence dictated seeding activity, we tested if it might also control strain composition. We previously developed the DS1 biosensor cell line, which expresses tau RD containing two disease-associated mutations (P301L and V337M) fused to YFP (4). Although it represents a rarified experimental system, this line has proved very useful because it readily propagates myriad tau strains. Depending on their replication efficiency, some strains propagate faithfully, whereas others will sector, *i.e.,* inclusions will steadily disappear from the cell population. Thus, one simple and robust classifier of strain identity is the ability to maintain itself in dividing cells, or to sector.

**Figure 3:**
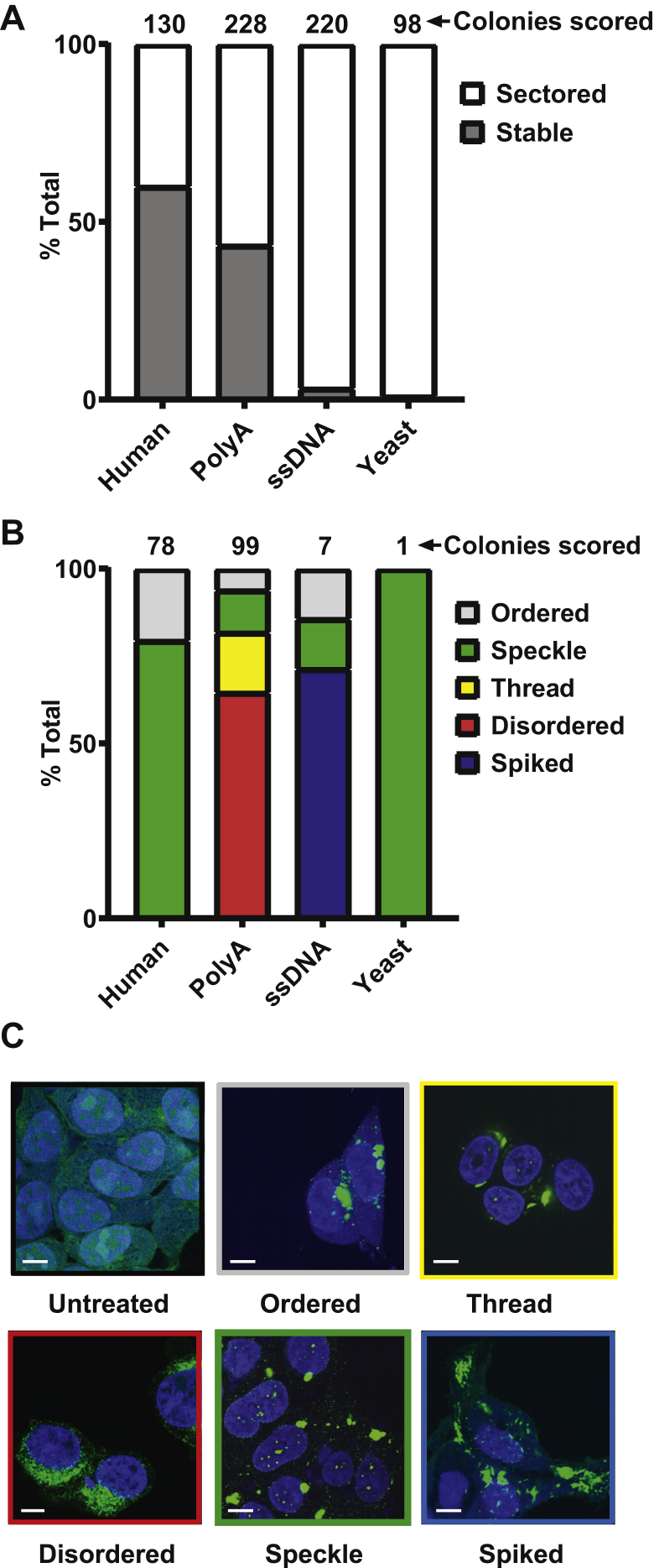
**RNA inducer dictates aggregate maintenance and strain identity.***A*, Tau seeds derived from different nucleic acid sources were transduced into DS1 cells, and single cells were sorted into 96-well plates and monitored over time. Certain nucleic acids (human, polyA) created more stable strains than others (ssDNA, yeast), which lost aggregates or sectored almost completely. N = 130 derived from human RNA, 228 from polyA, 220 from DNA, and 98 from yeast RNA. *B*, stable colonies containing inclusions were imaged using an In Cell Analyzer 6000, and resulting images were scored for morphology, blinded to the method of seed induction. Distribution of morphologies varied by inducer. N = 78 derived from human RNA, 99 from polyA, and 7 from ssDNA, 1 from yeast. *C*, exemplars of morphologies. Scale bar = 6 μm.

Tau was incubated in the presence of total human RNA, total yeast RNA, polyA RNA, or ssDNA before treating DS1 cells with induced tau seeds to initiate inclusion formation. After 72 h, we used fluorescence activated cell sorting to isolate 384 single aggregate-containing cells for each condition in the individual wells of 96-well plates (which was possible based simply on gating for the high fluorescence intensity that occurred when RD-YFP aggregates (8)). After 2 weeks of cell growth, we counted amplified colonies (n = 130 derived from human RNA, n = 228 from polyA, n = 220 from DNA, n = 98 from yeast RNA) that still propagated aggregates or had sectored. Sixty percent of colonies induced by human RNA:tau and 43% of colonies induced by polyA:tau still contained inclusions, while only 1% of colonies induced by yeast RNA:tau and 3% of colonies induced by ssDNA:tau contained inclusions (Fig. 3*A*). Hence, despite starting with 100% inclusion-bearing cells derived from tau seeds, across multiple inducers, human RNA most efficiently induced conformations of tau that created stable strains.

To further explore the effect of RNA origin on strain identity, we maintained remaining stable colonies (n = 78 human RNA, 99 polyA RNA, 7 ssDNA, and 1 yeast RNA) for characterization of inclusion morphology, a surrogate for strain identity (4). Colonies were assessed *via* fluorescence microscopy and blindly scored for morphology. After unblinding and quantification, human RNA–derived colonies largely had speckle morphology (79%), while remaining colonies were ordered (21%) (Fig. 3, *B* and *C*). Colonies derived from polyA RNA were primarily disordered (65%), in addition to thread (17%), speckle (12%), and ordered (6%) morphologies (Fig. 3, *B* and *C*). While relatively fewer ssDNA colonies stably maintained aggregates, we observed a distinct spiked morphology in 71% of colonies and additional colonies with speckle (14.5%) and ordered (14.5%) patterns (Fig. 3, *B* and *C*). The sole remaining yeast RNA–derived colony produced speckle morphology (Fig. 3, *B* and *C*). Although distinct inducers of intracellular aggregates generally displayed a heterogenous profile of intracellular morphologies, the predominant morphology for each inducer was unique.

### Soluble AD seeds are stabilized by RNA

**Figure 4:**
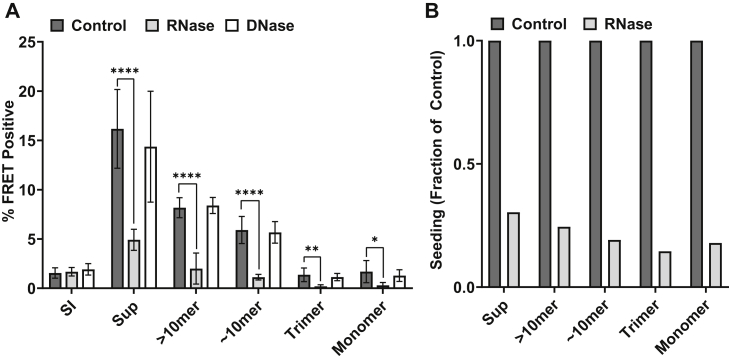
**RNA stabilizes soluble seeds from AD brain.** Homogenate from AD brain was fractionated in sarkosyl *via* ultracentrifugation. The supernatant (sup) was further fractionated using SEC. *A*, resultant fractions were treated with RNase, DNase, or vehicle for 24 h and seeded onto v2L biosensors. The percentage of FRET-positive cells was determined at 48 h. Soluble AD seeds (Sup, >10mer, ∼10mer, trimer, monomer) were highly sensitive to RNase (Sup, >10mer, ∼10mer *p* < 0.0001, trimer *p* = 0.0025, monomer *p* = 0.015, multiple unpaired *t* tests as compared to control) but not DNase (Sup *p* = 0.54, >10mer *p* = 0.68, ∼10mer *p* = 0.74, trimer *p* = 0.49, monomer *p* = 0.45, multiple unpaired *t* tests as compared to control). Sarkosyl insoluble (SI) AD fibrils were unchanged in all conditions (*p* = 0.65 RNase *versus* control, *p* = 0.28 DNase *versus* control). Graphs represent data from two experimental replicates, each performed in technical triplicate. These experiments are representative of similar studies performed 12 times overall. Error bars = S.D. *B*, data from A normalized to untreated control seeding (% FRET positive). AD, Alzheimer’s disease; FRET, fluorescence resonance energy transfer; SEC, size-exclusion chromatography. ∗∗∗∗*p* < 0.0001, ∗∗∗*p* < 0.001, ∗∗*p* < 0.01, ∗*p* < 0.05.

Given the ability of RNA to facilitate tau seeding *in vitro* and strains in cells, we tested its stabilization of seeds derived from the most common tauopathy, Alzheimer’s Disease (AD) (29). We homogenized frontal lobe tissue from an AD patient and fractionated the tau based on sarkosyl-insoluble material, which produced fibrils (Fig. S3) and soluble fractions. We used size exclusion chromatography to resolve soluble assemblies of different sizes. Because each sample had different amounts of seeding activity, to ensure that we evaluated seeding accurately across samples, we first titrated each to empirically determine a dose-response curve and be sure we used extract amounts within the linear range (Fig. S4). We then exposed each fraction to RNase, DNase, or buffer. After 24 h, we quantified seeding activity using biosensor cells. In total and soluble fractions, we observed a ∼70 to 85% reduction in seeding after RNase treatment (Fig. 4). By contrast, fibrillar tau exhibited no change in seeding in presence of RNase. DNase had no effect on seeding for any fraction. These data indicated that soluble seeds in AD require RNA for stability.

## Discussion

This study has examined the role of RNA in the development and maintenance of tau seeds and strains *in vitro* and in AD. This preliminary work provides a conceptual framework for further tests of the origins of strains in humans. We initially found that tau bound multiple forms of single-stranded RNA with relatively high avidity, yet formation of seeds and fibrils depended on RNA sequence and size, with a maximally effective size >40 nt. RNA was critical to maintaining induced seed integrity *in vitro*. While RNA from a variety of sources induced seed-competent tau, when this was used to transduce a human biosensor cell system, strain stability was far more robust when seeds had been produced by induction with human RNA. Finally, soluble tau seeds from AD brain were highly sensitive to RNase but not DNase, indicating a critical role for RNA in the maintenance of AD-derived seeds. While more studies are required, these observations suggest that certain RNAs might specifically trigger the formation and maintenance of unique tau assemblies or strains, contributing to the diversity of tauopathies.

### RNA pathways, tau, and prions

Our finding that RNA modulates the conformation of tau assemblies builds on many prior reports that have linked tau and RNA-binding proteins (RBPs) in disease (reviewed in (30, 31). Tau has been reported to bind ribosome components, including rRNAs (32, 33, 34, 35, 36, 37), and can affect ribosome biogenesis and mRNA translation ((35, 38, 39, 40). Tau binds RBPs, such as TIA1 and SRRM2, that modulate RNA splicing and stress granule formation (20, 41, 42, 43, 44, 45, 46, 47, 48, 49, 50, 51, 52). Finally, tau directly binds RNA in inert (53) and seed-competent conformations (20). Our findings suggest that beyond simple charge-based structural or scaffold effects, the tau:RNA complex may underlie physiological and pathophysiological processes based on conversion of tau between inert and seed-competent states.

Like tau, the prion protein may also use RNA as a cofactor to replicate unique structures (reviewed in (54). RNA facilitates prion protein conversion *in vitro*, maintains infectivity in established prion strains, and acts in a strain-specific fashion (55, 56, 57, 58, 59, 60, 61, 62). Thus, our observations for tau may reflect a more general role for prions in regulating RNA or vice versa.

### RNA binding *versus* seed induction

We observed that tau binds single-stranded RNA independent of sequence, as avidities were similar for three homopolymeric RNAs. Given lack of knowledge about the binding mode of RNA to tau, we cannot refer to precise affinities, although the association/dissociation kinetics were most consistent with two RNA binding sites per tau molecule. Most studies involving tau and RNA have suggested nonspecific associations based on simple charge interactions (16, 17, 63, 64). One study suggested a preference for tRNA binding to tau overexpressed in cells (53). We observed that despite relatively generic binding properties, only some single-stranded forms of RNA induced tau assembly and/or seed formation, while other single-stranded nucleic acids and all double-stranded sequences did not. While ssDNA induced seeding, DNA exists largely as a double strand in cells, and dsDNA failed to induce tau fibrilization. This is consistent with prior reports (63, 65). Other work has described polyU induction of electron paramagnetic resonance signatures of in-register β-strands or increased ThT signal in tau (65, 66). In these studies, polydisperse RNA was incubated with repeat domain fragments (K18, K19) or mutated tau (P301L), which differs from the conditions used here, which were based exclusively on FL tau (2N4R) without disease-associated mutations or strong intrinsic self-assembly characteristics (67). In summary, despite evidence that many forms of RNA bind tau and induce seeding and fibril formation, this work is the first to highlight the role of RNA as a specific inducer and stabilizer of tau seeds and strains that propagate in cells.

### RNA:tau and seed formation

While all ratios of polyA and tau generated seeds, 2:1 produced the most efficient seeding *in vitro*. This agreed with the 2:1 best fit of our binding data. The biphasic response of tau seeding to RNA concentration agreed with a prior report of optimal protein:inducer stoichiometry in the context of tau polymerization in presence of heparin and arachidonic acid (68). RNAs did not induce tau seeding equally. We observed a minimum of 40 nt to induce measurable seeding in sensitive biosensors. Despite evidence of relatively nonspecific binding, which might have been mediated primarily by complementary charge interactions, RNA exerted a specific effect on tau seed formation and especially on the induction of stable tau strains.

### RNA induction of strains

We observed that virtually all tau in ordered assemblies (whether derived from heparin, DNA, or RNA) seeded acutely into biosensor cells. However, only certain RNAs preferentially formed structures that propagated faithfully over multiple cell divisions, *i.e.,* with strain characteristics. Importantly, in this study, we did not isolate and characterize individual strains as we have done previously (4, 6, 8), which is highly labor-intensive. However, we did characterize strains using the coarse-grain characteristics of stability and inclusion morphology and observed clear differences in strain composition based on the RNA inducer. We have previously observed that heparin-induced fibrils trigger the formation of strains relatively inefficiently. In our original study, we observed that only about 1 to 2% DS1 cells transduced by heparin:tau stably propagated a strain (4).

This work puts these findings in a new light. Specifically, we found that the source of inducer used to create tau seeds had an enormous impact on the stability of the strains that subsequently propagated in cultured HEK293T cells. For example, when we used heparin or yeast RNA, we readily formed fibrils and seeds that induced aggregation acutely in cultured cells, but inclusions did not persist. By contrast, induction of tau with human RNA efficiently triggered the formation of more stable strains. Coupled with data which indicated that RNA played a critical role in stabilizing induced fibrils and soluble AD seeds, these data suggested a role for RNA within the cell in the stabilization of strains. We hypothesize that strain stability in a cell will depend on the ability to bind specific classes or even sequences of RNA.

**Figure 5:**
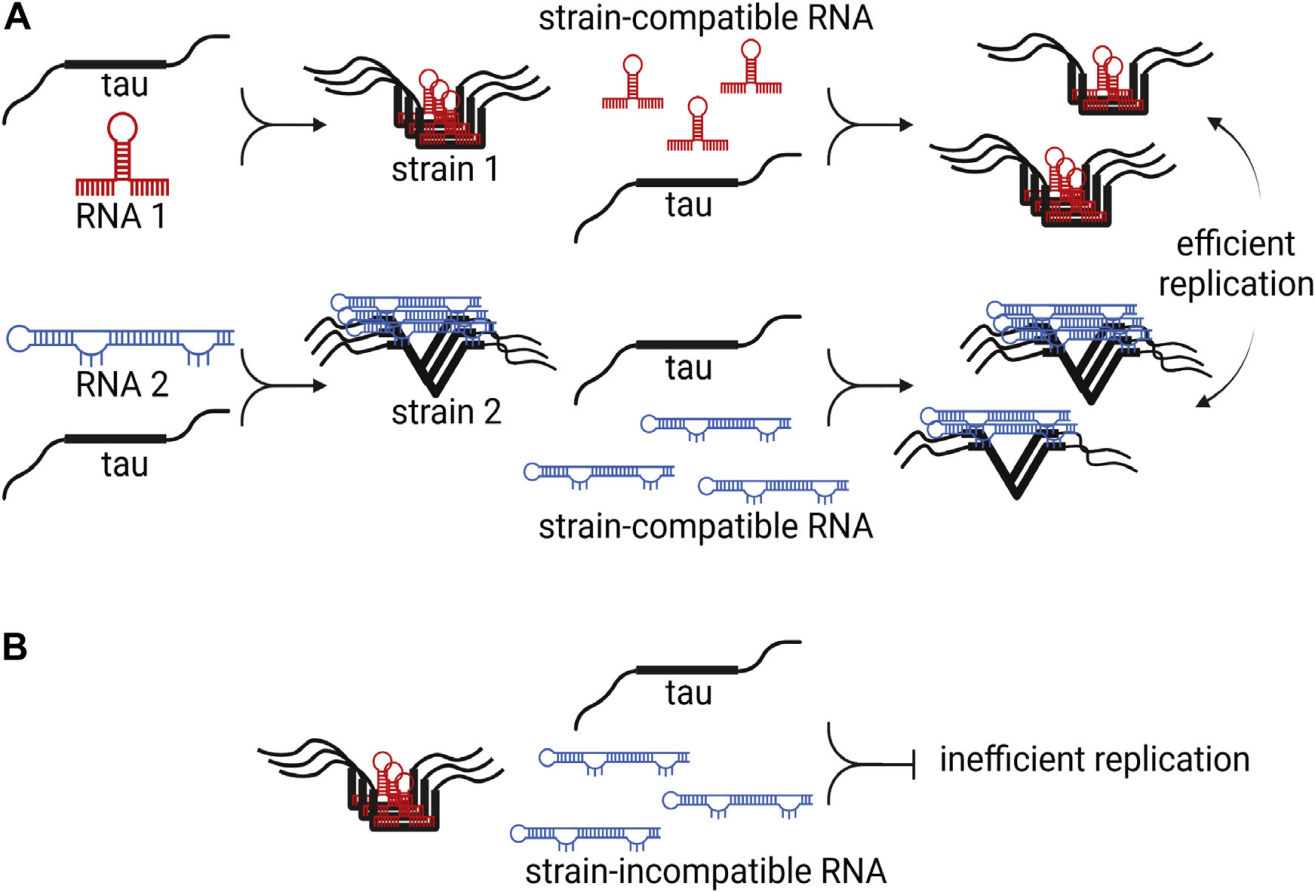
**Model of RNA:tau seeding and strain propagation.***A*, RNA serves as a sequence-specific inducer of tau seeds and strains. Stable cellular replication of a given strain depends on compatible RNA to be present as a co-factor. *B*, in the absence of compatible RNA, a given RNA-induced strain will replicate inefficiently.

According to this model (Fig. 5), yeast RNAs do not form stable strains in HEK293T cells because the conformations of tau induced by these molecules do not encounter suitable RNA sequences within the cell, and thus, the strains are rendered unstable. By contrast, use of human RNA as an inducer *in vitro* creates a tau conformation competent to bind similar RNAs within the cell and thus more stably propagates strains. These findings are consistent with the observation that RNA is associated with neurofibrillary tau deposits in human brain (18, 19). An intriguing extension of this hypothesis is that rather than simply recruiting RNA in disease states, tau might play a regulatory role in RNA metabolism. This idea will require much more detailed study but makes very specific predictions. First, RNA-dependent tau seeding activity should be found in normal conditions, and additionally, specific strains will bind unique classes or sequences of RNA that may in turn be required for their formation and/or stable maintenance.

### RNA stabilizes soluble seeds from AD brain

Consistent with our observations that RNA stabilized tau seeds *in vitro*, we observed that RNase treatment profoundly diminished the seeding activity of all forms of soluble tau from AD brain, whereas DNase treatment had no effect. This implied that seed-competent tau detected in the biosensor cell system requires RNA to maintain its activity. Despite a documented association with RNA (18, 19), seeding activity of detergent-insoluble tau was unaffected by RNase treatment. This could be due to maturation of seeds into a fibrillar conformation that no longer requires RNA or the contribution of other factors to fibril stability. Interestingly, it was recently reported that seeded soluble tau oligomers, and not fibrils, associate with RBPs, while fibrils are shuttled for autolysosomal degradation, suggesting unique physiological activity for oligomers (69). While we cannot exclude RNA as simply a nonstructural “co-factor” required for seeding, taken together with *in vitro* studies, these data suggest a primary role for RNA in converting and maintaining tau in a seed-competent form in AD.

## Conclusion

These experiments have explored the role of RNA in the conversion of tau from an inert to a seed competent form, the formation of unique strains, and in the stability of pathogenic assemblies, including soluble seeds in AD. Whether specific RNAs bind AD seeds, and whether they govern initial transformation of tau from an inert to a seed-competent form demand further study but could shed light on the mechanistic origins of tauopathy.

## Experimental procedures

### Cell culture

All cells were grown in Dulbecco’s Modified Eagle Medium (Gibco) supplemented with 10% fetal bovine serum (HyClone) and 1% Glutamax (Gibco). Cells were maintained at 37 °C, 5% CO_2_, in a humidified incubator and routinely tested for *mycoplasma* (VenorGem, Sigma).

### Tau expression and purification

We prepared FL wildtype (WT) tau (2N4R) protein as previously described (25).

### Nucleic acid purification

Total human RNA fractions were purified from HEK293T cells using Zymo Direct-zol RNA miniprep plus kits. Total yeast RNA (*Saccaromyces cerevisiae*) was purchased from Sigma. All nucleic acid preparations were stored in diethylpyrocarbonate treated H_2_O at −80 °C. All ssRNA and ssDNA sequences were synthesized by Sigma.

### Synthetic nucleic acid sequences

All ssRNA and ssDNA sequences were synthesized by Sigma. RNA and DNA was synthesized using phosphoramidite chemistry, followed by desalting and reverse-phase cartridge purification. Sequence accuracy and purity was verified by mass spectrometry and chromatography (Table 2).

**Table 2:** RNA and DNA sequences used

Synthetic RNA and DNA sequences used	Name	Sequence	nt length	MW
RNA	PolyA	AAAAAAAAAAAAAAAAAAAAAAAAAAAAAAAAAAAAAAAA	40	13,106
	PolyU	UUUUUUUUUUUUUUUUUUUUUUUUUUUUUUUUUUUUUUUU	40	12,185
	PolyC	CCCCCCCCCCCCCCCCCCCCCCCCCCCCCCCCCCCCCCCC	40	12,145
	Seq1	ACCGGGCGGAAACACCAACCGGGCGGAAACACCAACCGGG	40	12,978
	Seq2	AAACCCCAUAAACACCAAACCCCAUAAACACCAAACCCCCA	40	12,628
	Seq3	CGGGCGGCGGGGGGGCCCGGGCGGCGGGGGGGCCCGGGCG	40	13,266
DNA	Poly dA	AAAAAAAAAAAAAAAAAAAAAAAAAAAAAAAAAAAAAAAA	40	12,466
	Poly dT	TTTTTTTTTTTTTTTTTTTTTTTTTTTTTTTTTTTTTTTT	40	12,106
	dSeq1	ACCGGGCGGAAACACCAACCGGGCGGAAACACCAACCGGG	40	12,338
	dSeq2	AAACCCCATAAACACCAAACCCCATAAACACCAAACCC	40	12,015
	dSeq3	CGGGCGGCGGGGGGGCCCGGGCGGCGGGGGGGCCCGGGCG	40	12,626
	ssDNA Seq1	GTTAAGTCCCGAACTAGATGTGACCTAACGGTAAGAGAAT	40	12,377
	ssDNA Seq1′	CAATTCAGGGCTTGATCTACACTGGATTGCCATTCTCTTA	40	12,377

Abbreviations: nt, polynucleotide; MW, molecular weight.

### Fibrillization

FL WT tau monomer was filtered through a 100 kDa molecular weight cut-off filter (Corning) as instructed by the manufacturer (15,000*g* for 15 min). Filtered protein was collected, and protein concentration determined *via* DeNovix DS-11 Spectrophotometer. RNA or DNA was boiled for 7 min and snap-cooled on ice before addition to filtered monomer at determined mass ratio. All nucleic acid concentrations were quantified on a DeNovix DS-11 Spectrophotometer. Tau monomer was brought to a final concentration of 8 μM using tau buffer (10 mM Hepes, 100 mM NaCl in Millipore H_2_O). DTT was added at a final concentration of 11% before adding inducer (RNA, DNA, heparin) or tau buffer. Fibril mixtures were agitated at 350 rpm using a Thermomixer (Eppendorf) set to 37 °C for determined time points.

### Seeding assay

Stable v2L biosensor cells (24), which express tau repeat domain containing the P301S mutation, fused to cerulean or mClover fluorescent proteins, were plated at a density of 30,000 cells/well in a 96-well plate. After 18 h, at 60% confluency, cells were transduced with tau fibrils using Lipofectamine 2000 (Invitrogen). Cells were incubated with tau fibrils for 48 h before harvesting for flow cytometry.

### Flow cytometry

Transduced biosensor cells were harvested with 0.05% trypsin and fixed in 2% paraformaldehyde for 10 min before being resuspended in flow cytometry buffer (Hank’s Balanced Salt Solution supplemented with 1% fetal bovine serum and 1 mM EDTA) and evaluated by flow cytometry (Fortessa, GE) as described previously (23, 24). Three technical replicates were used for each dataset. For each experiment, a minimum of 5000 single cells per replicate were analyzed. Data analysis was performed using FLOWJO v10 and GraphPad PRISM v9.

### Strain-containing monoclonal generation

Stable cell lines expressing tau-repeat domain with P301L/V337M mutation fused to YFP (DS1) (4) were plated at a density of 25,000 cells/well in 96-well plates. Tau fibrils generated from RNA or DNA were transduced into cells using Lipofectamine 2000 and allowed to incubate for 72 h. Cells were then trypsinized and resuspended in flow buffer before live-cell sorting. Aggregate-containing cells were identified by their bright YFP signal using a fluorescence activated cell sorting Aria II SORP cell sorter, as previously described (8). Cells were sorted individually into a 96-well plate and grown until confluency to derive monoclonal lines that propagated tau strains.

### Aggregate maintenance assay

Sorted monoclonal colonies grew in 96-well plates for 2 weeks before being scored and amplified. After 2 weeks, viable cell colonies containing aggregates were monitored for 2 months and scored as maintaining aggregates or as being sectored (loss of all aggregates). Colonies were passaged into 24-well plates and then 12-well plates before storage in liquid nitrogen.

### Fixed cell imaging

Stably passaged monoclonal cell lines were plated onto a Corning Special Optics 96-well plate and grown to ∼40% confluency before fixing in 4% paraformaldehyde, permeabilizing in 0.1% Triton-X 100, and staining with DAPI. Cells were then imaged for GFP (488 nm) and DAPI (405 nm) *via* confocal microscopy (IN CELL Analyzer 6000, GE). Each colony was imaged from 13 distributed points in each well, across 5 z-stacks. IN CELL software then compiled maximum intensity projections across z-stacks to create a single image from each point scanned. Maximum intensity projection files were coded and blindly scored for tau aggregate morphology before unblinding and quantification.

Exemplar colonies were plated onto Ibidi 8 well μ-slides (cat no. 80826) and grown to ∼40% confluency before fixing in 4% paraformaldehyde, permeabilizing in 0.1% Triton-X 100, and staining with DAPI. The fixed cells were then imaged for GFP (488 nm) and DAPI (405 nm) on a confocal microscope (Nikon CSU-W1) at 100X objective using immersion oil. Images were acquired using an Orca-Fusion sCMOS camera (Hamamatsu) with the NIS Element Software.

### Transmission electron microscopy

Fibrils (4 μM) were deposited onto glow-discharged Formvar-coated 400-mesh copper grids for 30 s, washed with distilled water, and then negatively stained with 2% uranyl acetate for 1 min. Images were acquired on a Tecnai G^2^ spirit transmission electron microscope (FEI), serial number: D1067, equipped with LaB_6_ source at 120 kV using a Gatan ultrascan CCD camera.

### Biolayer interferometry

Second Generation Amine Reactive Biosensors (AR2G, ForteBio) were incubated with EDC and Sulfo-NHS to activate carboxylic acids. FL WT tau was diluted in acetate (pH 6) to charge primary amines and allow immobilization on biosensors. After quenching biosensors with ethanolamine, tau-coated sensors were dipped into serial 3-fold dilutions (1/2 log) of RNA to determine association and disassociation kinetics. Data from wells containing no RNA and biosensors containing no tau were background subtracted. Data were analyzed using Octet Data Analysis software using y-axis alignment, Savitz-Golay filtering, and interstep correction.

### Enzyme treatment

Pre-formed tau fibrils were incubated with a mixture of RNase A (3.2 mg/ml, Thermo) and RNase T1 (316 U/μl, Thermo), DNase I (633 U/μl, NEB), Heparinase I (3797 U/ml), or Millipore H_2_O (Control) and were agitated at 750 RPM in a Thermomixer (Eppendorf) set to 37 °C for 24 h.

### Human brain homogenization

0.5 g sections of frontal lobe from AD brain were dounce homogenized in PHF buffer (10 mM Tris-HCl [pH 7.4], 0.8 M NaCl, 1 mM EGTA, and 10% sucrose) (70) and sarkosyl insoluble fractions prepared as previously described (71) using ultra-centrifugation at 186,000*g* for 1 h at 4 °C to separate pellet from supernatant. Supernatant was flash frozen and stored at −80 °C for subsequent size fractionation. Pellets were washed with PBS for 30 min at 186,000*g*, 4 °C and resuspended in 50 mM Tris-HCL (pH 7.5) before flash freezing and storing at −80 °C.

### Size-exclusion chromatography

AD ultra-centrifuged supernatant (1 ml) was loaded onto a Superdex 200 Increase 10/300 Gl Column (GE Healthcare) and eluted in PHF buffer. Fractions were concentrated using 2000kD molecular weight cut-off filters (Vivacon), evaluated for protein concentration using A215 spectra, flash frozen in liquid nitrogen, and stored at −80 °C. Aliquots were thawed immediately before use in biochemical and seeding assays. The molecular weight/radius of proteins in each fraction was estimated by running gel filtration standards (Bio-Rad): Thyroglobulin (bovine) 670 kDa/8.5 nm; γ-globulin (bovine) 158 kDa/5.29 nm; ovalbumin (chicken) 44 kDa/3.05 nm; myoglobin (horse) 17 kDa/2.04 nm; and vitamin B121.35 kDa/0.85 nm. In a prior publication (71), Figure 1*E*, we demonstrated through use of crosslinking with SDS-PAGE that the size-exclusion chromatography protocol used in this work reliably purifies monomer, dimer, and trimer.

## Data availability

All data generated and analyzed during this study are included in this article.

## Supporting information

This article contains supporting information.

## Conflict of interest

The authors declare that they have no conflicts of interest with the contents of this article.

## Supporting information

Supplemental Figure 1 **Additional binding curves**. BLI with tau immobilized onto AR2G biosensors and exposed to increasing concentrations of polyU RNA or ssDNA Seq1 (half-log dilutions: 41 μM, 13 μM, 4 μM, 1.3 μM, 0.4 μM, and 0.13 μM). Figure shows representative data of polyU RNA or ssDNA Seq1 binding to BLI AR2G sensors loaded with tau. K_DAPP_ calculated as mean of three independent experiments. Binding (nm) refers to the perturbation in reflected light from the biosensor. *Red lines* represent curves fit to primary data. (See also Fig. 1*A* and Table 1)

Supplemental Figure 2 **Time course of nucleic acid seeding**. *A*, tau monomer was incubated 1 week with nucleic acid before transduction of v2L biosensors. We quantified intracellular aggregation as % FRET positive *via* flow cytometry. Specific nucleic acids induced seed-competency in tau (Poly A, ssRNA Seq3) while others did not (PolyU, PolyC, ssRNA Seq1-2, PolydA, PolydT, dSeq1-3). *B*, time course of tau incubation with different nucleic acids over time. *C*, heatmap of data in B. All conditions quantified in technical triplicate. Error bars = S.D. FRET, fluorescence resonance energy transfer.

Supplemental Figure 3 **SI extraction of tau fibrils from AD brain.** After dounce homogenizing AD brain, insoluble fibrils were fractionated using sarkosyl and ultracentrifugation. Fibrils were negative stained and imaged with TEM. *A*, fibrils, scale bar = 100 nm; *B*, scale bar = 50 nm. AD, Alzheimer’s disease; SI, sarkosyl insoluble.

Supplemental Figure 4 **Titration of AD seed fractions on v2L cells**. AD homogenate was fractionated in sarkosyl with ultracentrifugation. Supernatant was further fractionated using SEC. Increasing protein amounts of (*A*) sarkosyl insoluble (SI), (*B*) supernatant, or (*C*) ∼10mer were transduced into v2L cells, and intracellular seeding was quantified after 48 h. Data were fit with nonlinear least squares regression curves to determine linear range of protein to use for subsequent nuclease experiments. All data points represent technical triplicates. Error bars = S.D. AD, Alzheimer’s disease; SEC, size-exclusion chromatography.
