# Supplementary figures and images for: RNA induces unique tau strains and stabilizes Alzheimer’s disease seeds
Source: J Biol Chem. 2022 Jun 11;298(8):102132. doi: 10.1016/j.jbc.2022.102132 (PMC9364032; doi:10.1016/j.jbc.2022.102132)

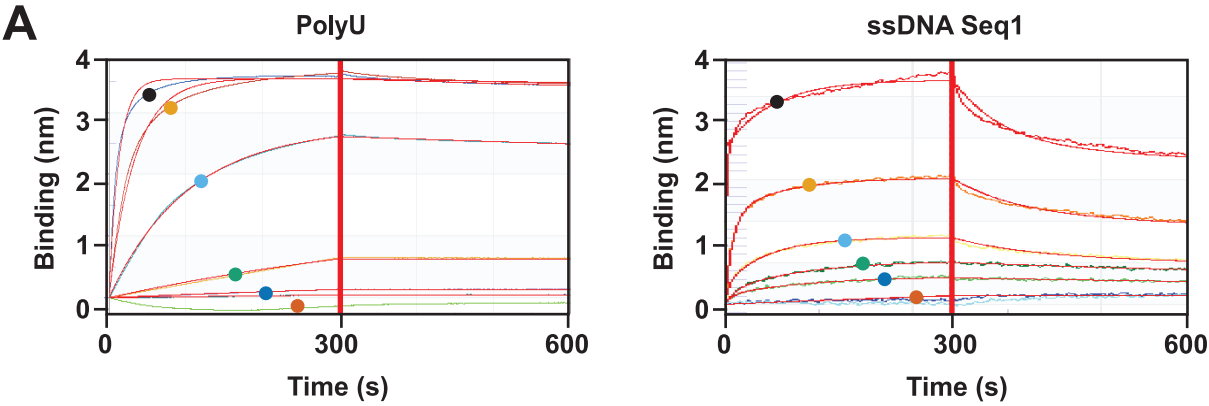

Supplement: Supplemental Figure 1 — Additional binding curves. BLI with tau immobilized onto AR2G biosensors and exposed to increasing concentrations of polyU RNA or ssDNA Seq1 (half-log dilutions: 41 μM, 13 μM, 4 μM, 1.3 μM, 0.4 μM, and 0.13 μM). Figure shows representative data of polyU RNA or ssDNA Seq1 binding to BLI AR2G sensors loaded with tau. KDAPP calculated as mean of three independent experiments. Binding (nm) refers to the perturbation in reflected light from the biosensor. Red lines represent curves fit to primary data. (See also Fig. 1A and Table 1) [file mmc1.pdf]

**A**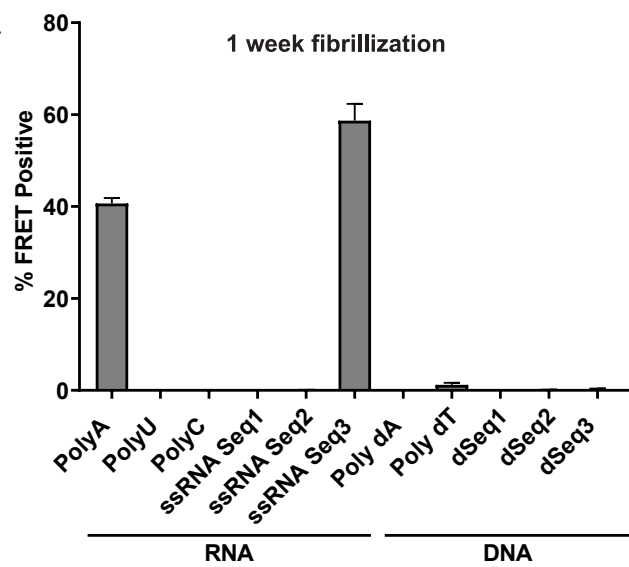**B**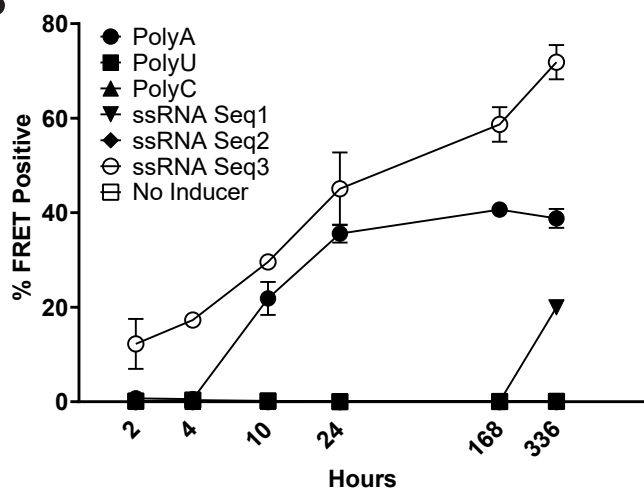**C**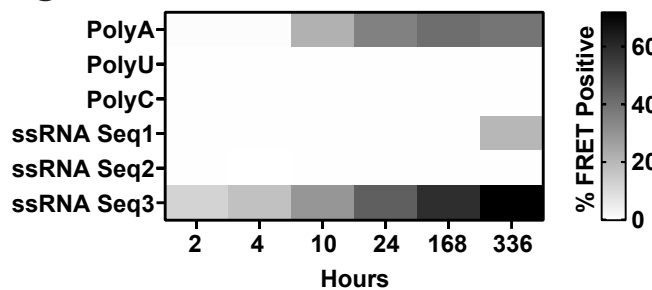

Supplement: Supplemental Figure 2 — Time course of nucleic acid seeding. A, tau monomer was incubated 1 week with nucleic acid before transduction of v2L biosensors. We quantified intracellular aggregation as % FRET positive via flow cytometry. Specific nucleic acids induced seed-competency in tau (Poly A, ssRNA Seq3) while others did not (PolyU, PolyC, ssRNA Seq1-2, PolydA, PolydT, dSeq1-3). B, time course of tau incubation with different nucleic acids over time. C, heatmap of data in B. All conditions quantified in technical triplicate. Error bars = S.D. FRET, fluorescence resonance energy transfer. [file mmc2.pdf]

**A**

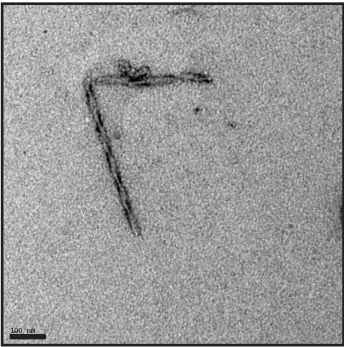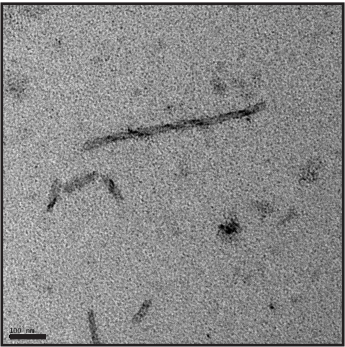

**B**

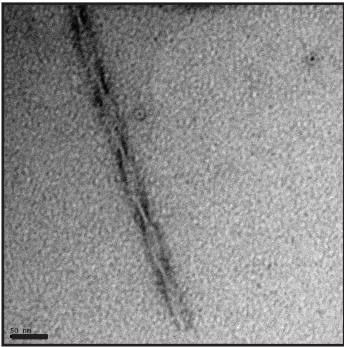

Supplement: Supplemental Figure 3 — SI extraction of tau fibrils from AD brain. After dounce homogenizing AD brain, insoluble fibrils were fractionated using sarkosyl and ultracentrifugation. Fibrils were negative stained and imaged with TEM. A, fibrils, scale bar = 100 nm; B, scale bar = 50 nm. AD, Alzheimer’s disease; SI, sarkosyl insoluble. [file mmc3.pdf]

**A**

SI

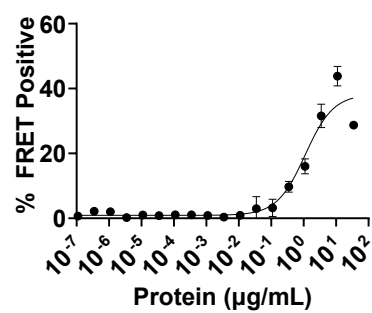**B**

Sup

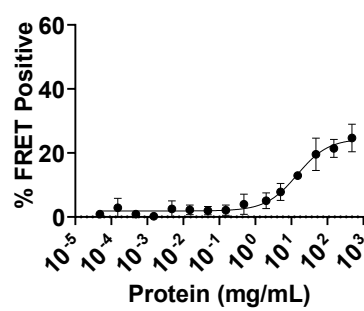**C**

~10mer

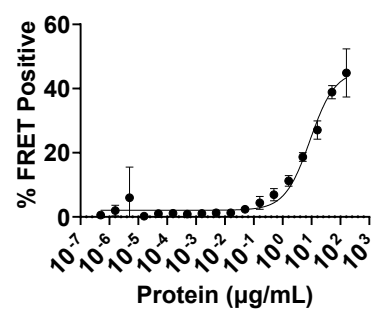

Supplement: Supplemental Figure 4 — Titration of AD seed fractions on v2L cells. AD homogenate was fractionated in sarkosyl with ultracentrifugation. Supernatant was further fractionated using SEC. Increasing protein amounts of (A) sarkosyl insoluble (SI), (B) supernatant, or (C) ∼10mer were transduced into v2L cells, and intracellular seeding was quantified after 48 h. Data were fit with nonlinear least squares regression curves to determine linear range of protein to use for subsequent nuclease experiments. All data points represent technical triplicates. Error bars = S.D. AD, Alzheimer’s disease; SEC, size-exclusion chromatography. [file mmc4.pdf]
